# Worldwide research productivity in the field of electronic cigarette: a bibliometric analysis

**DOI:** 10.1186/1471-2458-14-667

**Published:** 2014-06-30

**Authors:** Sa’ed H Zyoud, Samah W Al-Jabi, Waleed M Sweileh

**Affiliations:** 1 Poison Control and Drug Information Center (PCDIC), College of Medicine and Health Sciences, An-Najah National University, Nablus, Palestine; 2 Department of Clinical and Community Pharmacy, College of Medicine and Health Sciences, An-Najah National University, Nablus, Palestine; 3 WHO Collaborating Centre for Drug Information, National Poison Centre, Universiti Sains Malaysia (USM), Penang, Malaysia; 4 Department of Pharmacology and Toxicology, College of Medicine and Health Sciences, An-Najah National University, Nablus, Palestine

**Keywords:** Bibliometric, Electronic cigarette, E-Cigarette, Scopus

## Abstract

**Background:**

Electronic cigarette (EC) is an emerging phenomenon that is becoming increasingly popular with smokers worldwide. There is a lack of data concerning the evaluation of research productivity in the field of EC originating from the world. The main objectives of this study were to analyse worldwide research output in EC field, and to examine the authorship pattern and the citations retrieved from the *Scopus* database.

**Methods:**

Data were searched for documents with specific words regarding EC as “keywords” in the title. Scientific output was evaluated based on the methodology developed and used in other bibliometric studies by investigation: (a) total and trends of contributions in EC research during all previous years up to the date of data analysis (June 13, 2014); (b) authorship patterns and research productivity; (c) countries contribution; and (d) citations received by the publications.

**Results:**

Three hundred and fifty-six documents were retrieved comprising 31.5% original journal articles, 16% letters to the editor, 7.9% review articles, and 44.6% documents that were classified as other types of publications, such as notes or editorials or opinions. The retrieved documents were published in 162 peer-reviewed journals. All retrieved documents were published from 27 countries. the largest number of publications in the field of EC was from the United States of America (USA); (33.7%), followed by the United Kingdom (UK); (11.5%), and Italy (8.1%). The total number of citations at the time of data analysis was 2.277, with an average of 6.4 citations per document and median (interquartile range) of 0.0 (0.0–5.0). The *h*-index of the retrieved documents was 27. The most productive institutions were Food and Drug Administration, USA (4.2% of total publications) followed by Universita degli Studi di Catania, Italy (3.9%), University of California, San Francisco, USA (3.7%).

**Conclusions:**

This bibliometric study is a testament to the progress in EC research from the world over the last few years. More effort is needed to bridge the gap in EC-based research and to promote better evaluation of EC, risks, health effects, or control services worldwide.

## Background

Cigarette smoking is one of the leading health care problems in the world [1]. This is because cigarette smoking causes a broad range of diseases such as lung cancer, strokes, heart disease, chronic lung disease and other cancers, many of which are fatal. Smoking continues to be the most preventable cause of morbidity and mortality contributing to around half a million deaths every month, a situation that is likely to worsen in the future [1]. Electronic cigarette (e-Cigarette (EC)) is an emerging phenomenon that is becoming increasingly popular with smokers worldwide [2,3]. EC may be considered a lower risk substitute for factory-made cigarettes [4]. In addition, people report using them to reduce cigarette smoking consumption, to help quit smoking, and to relieve tobacco smoking withdrawal symptoms due to workplace smoking restrictions [5-7]. Little is known about EC, as few research reports have been published [6,8]. A recently published systematic review about e-cigarettes recommended that clinicians are advised to be aware that these devices are unregulated, of unidentified safety, and of doubtful benefit in quitting smoking [9].

Worldwide and during the last few years; several studies have measured and analysed the scientific research output [10-17]. In contrast, the evolution of scientific research output in the field of tobacco use has been poorly explored to date, and there are very few internationally bibliometric studies published within the field of tobacco use [18-24]. To the best of our knowledge, there is a lack of data concerning the evaluation of research productivity in the field of EC originating from the world.

Bibliometric analysis is a useful tool using specific indicators to obtain information about the current status of research in particular areas and allows researchers to identify and undertake new lines of research [25]. Bibliometric indicators involve the application of statistical methods to scientific publications to obtain the bibliographics for each country. These methods are mainly quantitative and are also used to make pronouncements about qualitative pictures of scientific activities [12,14-16,26]. Bibliometric indicators are useful tools for assessing scientific relevance of a given field and for appraising research output quality [12,14-16].

The objectives of this study were to analyse the worldwide research output in the field of EC, and to examine the authorship pattern and the citations retrieved from the Scopus database. A comprehensive online search was performed using SciVerse, Scopus, which is one of the world’s largest abstract and citation databases of peer-reviewed literature. Scopus contains 41 million records and covers nearly 18,000 titles from 5000 publishers worldwide, and provides 100% MEDLINE coverage [27]. This study will lead to better understanding of the current and future status of research in the field of EC. Furthermore, the results of this study will provide a general picture in the field of EC for researchers and clinicians to improve smoking research in the next decade.

## Methods

### Search strategy

The data used in this study were based on the Scopus online database. A comprehensive online search was performed using SciVerse, Scopus, which is one of the world’s largest a databases of peer-reviewed literature. Scopus covers nearly 18,000 titles from 5000 publishers worldwide, and contains 41 million records and provides 100% MEDLINE coverage [27]. Scopus database was developed by Elsevier, combining the characteristics of both Web of Science and PubMed. These characteristics allow for enhanced service for educational and academic needs, and medical literature research and bibliometric analysis. Scopus offers a basic search, or an advanced search. In the basic search, the results for the chosen keywords can be limited by the date of publication, subject area, and document type [28]. The search output from Scopus can be presented as a list of 20–200 items per page, and extracted documents can be exported to Microsoft Office Excel®. The results can be refined by document type, author name, source title, publications per year, and/or subject area, and a new search can be initiated within the results [28].

The key words entered in Scopus engine to achieve the objectives of this study were “electronic cigarette”, “e-cigarette”, “electronic vaping device”, “personal vaporizer”, and “electronic nicotin” as “Article Title”. All subject areas were selected for this research: health sciences, social sciences, life sciences, and physical sciences including all previous years up to the date of data analysis (June 13, 2014). The resultant search was as follows: Your query: TITLE (“electronic cigarette”) OR TITLE (e-cigarette) OR TITLE (“electronic vaping device”) OR TITLE (“personal vaporizer”) OR TITLE (“electronic nicotine”). We excluded documents that published as erratum or as chapter book. We also excluded those documents in which the primary focus was not a dimension of EC.

Scientific output was evaluated based on a methodology developed and used in other bibliometric studies [23,24,29-31]. The collated data were used to generate the following information: (a) total and trends of contributions in EC research during the time frame of research productivity; (b) authorship patterns and research productivity; (c) countries contribution; and (d) the citations received by the publications.

### Indices of research productivity

The measurements of bibliometric analysis (e.g. countries, cited articles, institutions) were converted to rank order using the standard competition ranking (SCR). Only the 10 top ranked were taken into consideration. If the measurements of bibliometric analysis have the same ranking number, then a gap is left in the following ranking numbers [24]. The *h*-index for data collected from Scopus is presented. The *h*-index is a country's number of articles (*h*) that have received at least *h* citations. It quantifies both country scientific productivity and scientific impact and it is also applicable to scientists and journals, etc. [32]. That is to say, a country with an *h-*index of 10 has published 10 documents, and each has attracted at least 10 citations. Documents with fewer than 10 citations are not calculated by the index. The *h*-index was originally developed as a way of qualifying research performance [33]. Two common performance indicators were considered for the top 10 ranked journals using data from the most recent year available [24,34]. First, the journal impact factor (IF) was evaluated using the Journal Citation Report (JCR; Web of Knowledge) 2012 science edition by Thomson Reuters (New York, NY, USA). The second measure of journal performance used in the current study was the *SCImago Journal Rank* (SJR) indicator. A detailed explanation on how the SJR calculation is made can be found on the *SCImago* website (available at: http://www.scimagojr.com/SCImagoJournalRank.pdf, Accessed June 13, 2014).

### Ethical approval

The Institutional Review Board (IRB) at An-Najah National University does not require submission of an IRB application for a bibliometric study. The IRB confirmed that there is no risk to human subjects in this type of research since the data are based on published literature and, as secondary data, did not involve any interactions with human subjects.

### Statistical analysis

Data from Scopus were exported to Microsoft Excel® and then transferred to the Statistical Package for Social Sciences, Version 15 (SPSS; SPSS Inc., Chicago, IL, USA) programme for analysis. Variables that were not normally distributed, such as the number of citations, are expressed as a median (Q1–Q3: interquartile range). Categorical data are expressed as numbers with percentages.

## Results

A total of 356 documents on EC were indexed in the Scopus database. Analysis of document types showed that original article type was the most-common (112;31.5%). The remaining documents were letter to the editor (57; 16%), reviews (28; 7.9%), short communications (27; 7.6%) and 132 documents that were classified as other types of publications (37%) such as notes or editorials or opinions. Of those original articles, 48 were relevant to population surveys; 27 were relevant to chemical analyses of samples of EC; and 37 were relevant to clinical trials designed to compare efficacy and safety of EC. The results of publication output are shown in Table 1. For the period from 2007 to 2014, the annual number of documents published indicates that EC research productivity was low in the first years but showed an obvious increasing in the recent years. The first document related to EC was published in 2007 and next documents was published in 2009 (Table 1). The main language in which the documents were published was dominated by English (317, 89%) followed distantly by German (17, 4.8%), and French (9, 2.5%). Table 2 shows research areas of interest pertaining to published documents in the field of EC. Medicine, as a research area, was the most common (313; 87.9%) followed by social sciences (42; 11.8%) and pharmacology/toxicology/pharmaceutics with 28 (7.9%) documents.

**Table 1:** Total articles included in bibliometric analysis in the field of electronic cigarette by publication year

**Year**	**Total N = 356 (%)**
2007	1 (0.3)
2009	6 (1.7)
2010	13 (3.7)
2011	26 (7.3)
2012	47 (13.2)
2013	116 (32.6)
2014	147 (41.3)

**Table 2:** The top 10 ranking of areas of interest of published articles associated with electronic cigarette

**SCR**^**a**^	**Areas of interest**	n (%)*
1st	Medicine	313 (87.9)
2nd	Social Sciences	42 (11.8)
3rd	Pharmacology, Toxicology and Pharmaceutics	28 (7.9)
4th	Environmental Science	17 (4.8)
5th	Biochemistry, Genetics and Molecular Biology	9 (2.5)
6th	Psychology	8 (2.2)
6th	Chemistry	8 (2.2)
8th	Nursing	7 (2.0)
9th	Agricultural and Biological Sciences	5 (1.4)
10th	Health Professions	3 (0.8)

*Abbreviation: SCR* Standard Competition Ranking.

^a^Equal areas of interest have the same ranking number, and then a gap is left in the ranking numbers.

*Total exceeds 100% as data are overlapping due to multidisciplinary interaction.

The retrieved documents were published in 162 peer-reviewed journals. Table 3 shows the ranking of the 10 top journals in which EC related articles were published. Thirty five documents (9.8%) were published in *Tobacco Control* whereas 16 (4.5%) were published in *BMJ online*, 14 (3.9%) were published in *Addiction*, and 14 (3.9%) were published in *BMJ Clinical Research Ed.* All journals from the top 10 journal titles had an official IF and were listed in the JCR 2012. Only one journal in the top 10 ranking journals had SJR <1.

**Table 3:** Ranking the top 10 journals from the total of 162 journals in which electronic cigarette related articles were published with their impact factors

**SCR**^**a**^	**Journal**	**Frequency (%)**	**SJR**	IF (2012)*
1st	*Tobacco Control*	35 (9.8)	1.619	4.111
2nd	*BMJ Online*	16 (4.5)	1.479	1.583
3rd	*Addiction*	14 (3.9)	1.755	4.746
3th	*BMJ Clinical Research Ed*	14 (3.9)	1.48	17.215
5th	*Nicotine and Tobacco Research*	9 (2.5)	1.233	2.477
5th	*American Journal of Preventive Medicine*	9 (2.5)	2.310	3.945
7th	*JAMA- Journal of the American Medical Association*	7 (2.0)	4.843	29.978
7th	*Lancet*	7 (2.0)	7.074	39.060
9th	*International Journal of Environmental Research and Public Health*	6 (1.7)	0.628	1.998
9th	*American Journal of Public Health*	6 (1.7)	1.738	3.930

*Abbreviations: SCR* Standard Competition Ranking, *SJR* SCImago Journal Rank, *IF* impact factor, *BMJ* British Medical Journal.

^a^Equal journals have the same ranking number, and then a gap is left in the ranking numbers.

*The impact factor was reported according to Institute for Scientific Information (ISI) journal citation reports (JCR) 2012.

All retrieved documents were published from 27 countries. Table 4 shows a list of ranking 10 countries whose researchers published the largest number of articles in the field of EC. When the data were analysed by country, the largest number of publications in the field of EC was from the United States of America (USA); (33.7%), followed by the United Kingdom (UK); (11.5%), and Italy (8.1%); (Table 4). In addition, the total number of citations at the time of data analysis (June 13, 2014) was 2.277, with an average of 6.4 citations per document and median (interquartile range) of 0.0 (0.0–5.0). The *h*-index of the retrieved documents was 27 (i.e. 27 documents had been cited at least 27 times at the time of data analysis (June 13, 2014)). The highest *h*-index was 22 for the USA, followed by 12 for the UK, 9 for Sweden, and 6 for each Greece, New Zealand, and Switzerland (Table 4).

**Table 4:** The top 10 ranking of the most productive countries that published the largest number of articles in the field of electronic cigarette from the world

**SCR**^**a**^	**Country**	**Number of documents (%)**	***h*-index**
1st	United States of America	120 (33.7)	22
2nd	United Kingdom	41 (11.5)	12
3rd	Italy	29 (8.1)	9
4th	Greece	14 (3.9)	6
5th	New Zealand	12 (3.4)	6
5th	Switzerland	12 (3.4)	6
7th	Australia	8 (2.2)	4
7th	Canada	8 (2.2)	3
7th	Germany	8 (2.2)	1
10th	South Korea	7 (2.0)	4
10th	Poland	7 (2.0)	4
10th	France	7(2.0)	1

*Abbreviation: SCR* Standard Competition Ranking.

^a^Equal countries have the same ranking number, and then a gap is left in the ranking numbers.

In Table 5, a list of the most cited articles is shown [5,6,35-42]. Table 6 presents a list of the 10 most productive authors in the field of EC; those authors have published at least eight articles. Moreover, Table 7 shows the top 10 most productive institutions in the field of EC. The most productive institutions were Food and Drug Administration, USA (4.2% of total publications) followed by Universita degli Studi di Catania, Italy (3.9%), University of California, San Francisco, USA (3.7%).

**Table 5:** Ranking the top 10 cited articles related to electronic cigarette worldwide

**SCR**^**a**^	**Authors with year of publication**	**Title**	**Source title**	**Cited by**
1st	Bullen et al. 2010 [36]	**Effect of an electronic nicotine delivery device (e cigarette) on desire to smoke and withdrawal, user preferences and nicotine delivery: Randomised cross-over trial**	*Tobacco Control*	103
2nd	Etter and Bullen 2011 [39]	**Electronic cigarette: Users profile, utilization, satisfaction and perceived efficacy**	*Addiction*	90
3rd	Etter 2010 [6]	**Electronic cigarettes: A survey of users**	*BMC Public Health*	71
4th	Vansickel et al. 2010 [42]	**A clinical laboratory model for evaluating the acute effects of electronic “cigarettes”: Nicotine delivery profile and cardiovascular and subjective effects**	*Cancer Epidemiology Biomarkers and Prevention*	68
5th	Cahn and Siegel 2011 [37]	**Electronic cigarettes as a harm reduction strategy for tobacco control: A step forward or a repeat of past mistakes?**	*Journal of Public Health Policy*	67
6th	Polosa et al. 2011 [5]	**Effect of an electronic nicotine delivery device (e-Cigarette) on smoking reduction and cessation: A prospective 6-month pilot study**	*BMC Public Health*	64
7th	Ayers et al. 2011 [35]	**Tracking the rise in popularity of electronic nicotine delivery systems (electronic cigarettes) using search query surveillance**	*American Journal of Preventive Medicine*	57
8th	Eissenberg et al. 2010 [38]	**Electronic nicotine delivery devices: Ineffective nicotine delivery and craving suppression after acute administration**	*Tobacco Control*	52
9th	Siegel et al. 2011 [40]	**Electronic cigarettes as a smoking-cessation tool: Results from an online survey**	*American Journal of Preventive Medicine*	46
10th	Trtchounian et al. 2010 [41]	**Conventional and electronic cigarettes (e-cigarettes) have different smoking characteristics.**	*Nicotine & tobacco research*	44

*Abbreviation: SCR* Standard Competition Ranking.

^a^Equal documents have the same ranking number, and then a gap is left in the ranking numbers.

**Table 6:** Ranking top 10 prolific authors who published in the field of electronic cigarette with their affiliations and publication patterns

**SCR**^**a**^	**Author**	**No. (%)**^**b **^**of publications**	**Affiliation**
1st	Polosa, R.	15 (4.2)	Universita degli Studi di Catania, Department of Internal and Emergency Medicine, Catania, Italy
2nd	Farsalinos, K.E.	12 (3.4)	Onassis Cardiac Surgery Centre, Athens, Greece
2nd	Caponnetto, P.	12 (3.4)	Universita degli Studi di Catania, Centro per la Prevenzione e Cura del Tabagismo (CPCT), Catania, Italy
4th	Etter, J.F.	11 (3.1)	Institute of Social and Preventive Medicine, Faculty of Medicine, Geneva, Switzerland
5th	Bullen, C.	10 (2.8)	National Institute of Health Innovation, School of Population Health, The University of Auckland, Private Bag 92019, Auckland 1142, New Zealand
6th	Romagna, G.	9 (2.5)	ABICH S.r.l, Biological and Chemical Toxicology Research Laboratory, Verbania, Italy
7th	Goniewicz, M.L.	8 (2.2)	Department of Health Behavior, Division of Cancer Prevention and Population Sciences, Roswell Park Cancer Institute, , Buffalo, New York, USA.
7th	Talbot, P.	8 (2.2)	University of California, Riverside, Department of Cell Biology and Neuroscience, Riverside, United States
7th	Russo, C.	8 (2.2)	Universita degli Studi di Catania, Department of Internal and Emergency Medicine, Catania, Italy
7th	Grana, R.A.	8 (2.2)	University of California, San Francisco, Center for Tobacco Control Research and Education, San Francisco, United States

*Abbreviation: SCR* Standard Competition Ranking.

^a^Equal authors have the same ranking number, and then a gap is left in the ranking numbers.

^b^Percentage of publications for each author by the total number of documents.

**Table 7:** Ranking the top 10 highly productive institutions in the field of electronic cigarette

**SCR**^**a**^	**Institutions**	**No. of documents (%)**
1st	Food and Drug Administration, USA	15 (4.2)
2nd	Universita degli Studi di Catania, Italy	14 (3.9)
3rd	University of California, San Francisco, USA	13 (3.7)
4th	Onassis Cardiac Surgery Centre, Greece	12 (3.4)
4th	Barts and The London Queen Mary’s School of Medicine and Dentistry,UK	12 (3.4)
6th	University of California, Riverside, USA	9 (2.5)
7th	Roswell Park Cancer Institute, USA	7 (2.0)
7th	Institute of Social and Preventive Medicine, Switzerland	7 (2.0)
7th	Johns Hopkins Bloomberg School of Public Health, USA	7 (2.0)
7th	Faculty of Medical and Health Sciences, School of Population Health, New Zealand	7 (2.0)

*Abbreviations: SCR* Standard Competition Ranking, *UK* United Kingdom, *USA* United States of America.

^a^Equal institutes have the same ranking number, and then a gap is left in the ranking numbers.

## Discussion

Usage of EC is increasing worldwide. However, few data were found about efficacy, safety and health impact of EC. Nicotine is a dangerous an addictive substance that should be handled with care, and previous data indicated that more than 0.5 gram of oral nicotine might kill a human adult [43]. Our work focused primarily on assessing impact in the field (i.e., through number of publications), the productivity of particular institutions or academic departments, the relative contribution of authors, and the utility of various journals that include EC literature, which is considered as a sub-area of the multidisciplinary field of tobacco control by using a bibliometric analysis. Bibliometric analysis includes a series of visual and quantitative procedures of the communication and utilization of literature to evaluate scientific publications. Bibliometric studies have been applied primarily to reveal the global trends of research within a given topic, field, institute, or country [16,44]. This study was limited to 356 documents extracted from Scopus, bearing article titles with terms related to EC and, therefore, cannot be generalised to the EC literature covered by other databases such as Google Scholar. Although the number of citations for each publication might differ from one search engine to another, Scopus search engine remains one of the best available databases for analysing and tracking citations and comparing citations to different research groups and different institutions [45]. A study that compared Scopus, Google Scholar, PubMed, and Web of Knowledge found that PubMed is considered an important resource for clinicians and researchers, while Scopus offers the capability for citation analysis and covers a wider journal range [28,45-47].

In the present study, bibliometric indicators were used to describe the worldwide scientific activity in the field of EC. Based on the authors’ knowledge, this is the first study to analyse the quantity and quality of EC-based research. Research activity in this field showed a promising rise in small number of countries. This paper also adds to the emerging bibliometric literature within tobacco research [19,21-24].

The USA was the most productive country with its researchers being authors in 33.7% of all articles. As it can be seen in our study, the behaviour of every country in scientific research output was different. Our study showed that some countries, such as USA, UK and Italy, have higher EC research productivity than the world remaining countries. This activity depends on population, socio-economic status or overall scientific activity of the country [48]. The ten most productive countries that have published in the field of EC includes many nations nearly similar to other scientific productivity rankings [49]. The total publications found in Scopus between 2007 and 2014 showed a yearly increase. Around 40% of publications were published in 2014; however, the number of scientific research productivity in this year may be increasing because it is still open for new journals issues. Despite that EC have been developed by Beijing SBT Ruyan Technologies and Development, Beijing, China; and are marketed by the Create Times Industrial & Trading, Shenzhen, China [50], only one study from China has been published which mainly focused on portrayal of electronic cigarettes on YouTube, without considering the safety and effectiveness of this product to be used as a cessation aid [51].

The first article related to EC was published as letter to editor in 2007 in *tobacco control* has raised important questions focused on the safety and effectiveness of this product to be used as a cessation aid and on the presence of peer-reviewed or scientific evidence that supported the claims of the manufacturer for EC [50]. Although, no scientific article in the field of EC has been published in 2008, the evolution of research in the field of EC has shown an obvious increasing since 2009. In addition, EC research productivity has followed the general evolution in scientific research productivity observed in the last decade and especially in the recent years [19,22]. 1n 2012, one of the most cited articles in the field of EC which was published in *tobacco control,* has still raised the same questions of the first study published in this field. The authors concluded that many questions about EC remain unanswered such as the confirmed safety profile of this product, including in long-term users, and the efficacy of this product tested by clinical trials [52]. Furthermore, the same issues regarding the safety and efficacy of this product as a smoking cessation aid are still rising in the literature published in 2013 [53-55]. A recent systematic review indicated that EC is by far a lesser harmful alternative to smoking. There is no tobacco and no combustion involved in EC use; therefore, regular vapors may avoid several harmful toxic chemicals that are typically present in the smoke of tobacco cigarettes. Indeed, some toxic chemicals are released in the EC vapor as well, but their levels are substantially lower compared with tobacco smoke, and in some cases (such as nitrosamines) are comparable with the amounts found in pharmaceutical nicotine products [56]. The authors concluded that a more research is needed in several areas, such as atomizer design and materials to further reduce toxic emissions and improve nicotine delivery, and liquid ingredients to determine the relative risk of the variety of compounds (mostly flavorings) inhaled [56].

To the best of our knowledge, this study is the first of its kind to obtain initial data regarding the publication and citation productivity in the EC field using Scopus database; a database that is being used to evaluate the performance of institutes and their members. This study is not without limitations, most of which are the same as those of bibliometric studies performed in other biomedical fields [23,24,29-31]. First of all, in the current study, we used Scopus criteria for including EC-related keywords. Articles published in non-Scopus-cited journals were not included, although they might contribute to scientific productivity in the field of EC. Another limitation is that some articles might not be included as they did not point out EC and related terms in their titles, however, these terms were mentioned throughout the text. Therefore, it is possible that the number of publications analyzed in this study might not exactly represent all EC-based research activity. Finally, it should be noted that the research output for certain institutions or authors could have been underestimated due to differences in the spelling of their English names across various articles. Therefore, such institutions or authors might have two or more profiles in Scopus because their names were written differently in various articles.

## Conclusion

In conclusion, this bibiliometric study is a testament to the progress in the worldwide EC research over the last few years. Conducting research on EC is feasible in some countries. Although, the present data reveal a promising rise and a good start for research activity in the field of EC, the quantity of EC-based research activity originating worldwide is still inadequate for most countries. The worldwide EC research output is far below from what is needed, and diverse areas of EC research are still primitive. More effort is needed to bridge the gap in EC-based research and to promote better evaluation of EC, risks, health effects, or control services worldwide.

## Abbreviations

SPSS: Statistical package for social sciences; EC: Electronic cigarette; SD: Standard deviation; ISI: Institute for scientific information; USA: United States of America; UK: United Kingdom; JCR: Journal citation report; IFs: Impact factors; Q1-Q3: Lower quartile – upper quartile; SCR: Standard competition ranking.

## Competing interests

The authors declare that they have no competing interests.

## Authors’ contributions

All authors were involved in drafting the article, and all authors approved the final version to be submitted for publication. SZ conceived of the study conception and design, organized and supervised the data collection, and provided analysis, interpretation, and writing. SA and WS participated in the study design, and provided critical revision of manuscript for important intellectual content.

## Authors’ information

Dr. Sa’ed H. Zyoud is an assistant professor of clinical toxicology/pharmacy and the head of a research group in the field of clinical toxicology, clinical pharmacology/ pharmacy, social pharmacy, pharmacoepidemiology and drug safety, and a bibliometric analysis. S.Z and the research group have published many articles in leading international and high reputation journals. The research group has also supervised many students in the fields of nursing, public health and pharmacy.

## Pre-publication history

The pre-publication history for this paper can be accessed here:

http://www.biomedcentral.com/1471-2458/14/667/prepub
